# The feasibility of Technology, Application, Self-Management for Kidney (TASK) intervention in post-kidney transplant recipients using a pre/posttest design

**DOI:** 10.1186/s40814-023-01417-9

**Published:** 2023-11-22

**Authors:** Tara O’Brien, Karen Rose, Brian Focht, Noor Al Kahlout, Tad Jensen, Kenzie Heareth, Uday Nori, Reem Daloul

**Affiliations:** 1 The Ohio State University College of Nursing, Newton Hall, 1585 Neil Ave, Columbus, OH 43210 USA; 2 The Ohio State University College of Education and Human Ecology, 152 PAES, 305 Annie and John Glenn Ave, Columbus, OH 43210 USA; 3 The Ohio State University College of Medicine, 300 West 10Th Avenue Suite 1150, Columbus, OH 43210 USA; 4 Division of Nephrology, Kidney and Pancreas Transplant Program, Allegheny General Hospital, Erie, PA 16505 USA

**Keywords:** mHealth technology, Kidney transplant recipients, Physical activity tracking, Dietary intake tracking, Self-management of care

## Abstract

**Background:**

Weight gain after a kidney transplant remains a major problem that can lead to adverse effects on morbidity and mortality. The posttransplant phase provides a window of opportunity to improve the engagement of self-management of care for lifestyle modifications for diet and physical activity. The purpose of our study was to (1) test the feasibility of recruitment, retention, and adherence for using the Technology, Application, Self-Management for Kidney (TASK) intervention in post-kidney transplant recipients (≥ 18 years of age) at baseline, 4, 8, and 12 weeks; and (2) estimate the preliminary effects of the TASK intervention in producing change over time for blood pressure (BP), weight, fruits/vegetable intake, fiber intake, sodium intake, self-efficacy to exercise, and perceived stress.

**Methods:**

This study used a 12-week pre/posttest design using to test the feasibility of the TASK intervention. We applied paired *t*-tests and McNemar’s test to compare the outcomes at weeks 4, 8, and 12.

**Results:**

We met our recruitment goal (*N* = 20) and found a 15% attrition rate (*n* = 3) at Week 12. Adherence rate among the study completers for recording daily food intake was 83–94% over the 12 weeks and for recording daily physical activity was 17–33% over the 12 weeks. We observed improvements over time for BP, weight, fruits/vegetable intake, fiber intake, and sodium intake; these differences were non-significant, although clinically important. We did find a significant difference from baseline to 12 weeks in weight reduction (*p* = 0.02), self-efficacy to exercise (*p* = 0.003), and perceived stress (*p* = 0.04).

**Conclusions:**

The data suggest the TASK intervention was feasible for kidney recipients to use and resulted in weight control, increased self-efficacy to exercise, and decreased perceived stress.

**Trial registration:**

ClinicalTrials.gov #:NCT05151445

## Key messages regarding feasibility

- *What uncertainties existed regarding the feasibility?* Advancements in consumer-based mobile health (mHealth) applications technology provide kidney recipients an opportunity to engage in diet and physical activity management skills. Moreover, health coaching is an effective tool for supporting behavior change and improving health outcomes for people living with chronic disease. Evidence is lacking for the feasibility of kidney recipients to use a consumer-based mHealth dietary app for recording daily dietary intake/physical activity coupled with health coaching skills.
- *What are the key feasibility findings?* We met our recruitment goal (*N* = 20) and found a 15% attrition rate (*n* = 3) at Week 12. Adherence rates for recording daily dietary intake were higher than for physical activity throughout the study. We found a large effect (baseline mean 61, vs.12 weeks mean 81 (95% CI [6.51, 27.6], *d* = 0.83, *p* = 0.003), in self-efficacy to exercise and a medium effect for weight reduction (baseline 190.1 ± 44.6 to 12 weeks 186.6 ± 47, (95% CI [ 0.88–9.08], *d* = 0.62, *p* = 0.02) and perceived stress (baseline mean 22 vs.12 weeks mean 19 (95% CI [0.16, 4.90], *d* = 0.55, *p* = 0.04) from baseline to 12 weeks.
- *What are the implications of the feasibility findings for the design of the main study?* The TASK intervention was well received by kidney recipients to enhance self-management for physical activity and dietary intake. We found the TASK intervention to be feasible, resulting in weight control, increased self-efficacy to exercise, and decreased stress. Future studies should explore behavioral factors that contribute as facilitators or barriers in using the intervention.

## Introduction

 Posttransplant survival is complex for kidney transplant recipients, and survivability depends on active engagement in self-management of care (e.g., taking medications, consuming a healthy diet, and engaging in physical activity) to prevent organ rejection [1]. Previous studies have indicated that kidney recipients (KRs) gain an average of 22 pounds during the first year posttransplant [2]. Weight gain and obesity are common after kidney transplant, often leading to the development of cardiovascular disease, posttransplant diabetes, and death [3]. The cause of weight gain in this population has many contributing factors, including a high-calorie diet, physical inactivity, decreased physical function, immunosuppressant therapy, steroid therapy, and stress, all of which contribute to poor cardiometabolic health [4]. Multiple factors affect weight gain, such as age, sex, race, and stress [5]. Additionally, dietary intake affects several cardiovascular risk factors after transplantation. One study found that participants who had a lower body mass index (BMI) consumed more fruits and vegetables than those with a higher BMI [6].

The posttransplant phase provides a window of opportunity to improve the engagement of self-management of care for lifestyle modifications (diet and physical activity) [7]. Few studies, however, have explored the behavioral indicators for engaging KRs in their daily self-care practice [8–11]. Advancements in consumer-based mobile health (mHealth) application technology provide KRs an opportunity to engage in diet and physical activity management skills. The engagement of these skills includes recording, reminding, alerting, and monitoring calorie intake, sodium intake, carbohydrate intake, weight, blood pressure (BP), and physical activity via real-time data [12]. Overall, evidence suggests that mHealth technology is useful as a low-intensity approach to conventional lifestyle modification management strategies [13]. Mobile health technology to promote lifestyle modification holds promise for providing KRs with a cost-effective strategy to manage diet and physical activity tracking in real-time.

Health coaching is an effective tool for supporting behavior change and improving health outcomes for people living with chronic disease [14]. Health coaching is the process where the participant determines their goals, works toward their goals, and self-monitors behaviors to increase accountability, all within the context of an interpersonal relationship with a coach [15]. However, evidence is lacking for the feasibility of KRs to use a consumer-based mHealth dietary app for recording daily dietary intake/physical activity coupled with health coaching skills. Kidney recipients are the ideal population to test the feasibility of the intervention by using a mHealth app + health coaching skills. Kidney recipients are known to experience high rates of cardiovascular disease. Therefore, KRs are required (but often nonadherent after transplant) to make lifestyle modifications (e.g., diet, exercise, weight control, and medication adherence) to prevent the loss of the transplanted kidney [16]. One such dietary and physical activity app that has successfully improved diet consumption and physical activity is a consumer-based mHealth app called Lose-It^©^ [17]. This consumer-based dietary app has the features to allow KRs to understand their dietary and physical activity patterns. Previously, we found that the Lose-It^©^ app was feasible for older women living in the Appalachian region with chronic disease to record their daily dietary intake [18].

Traditional behavioral change techniques (education) alone are not enough to support behavior change [19]. However, there is evidence that mHealth apps, using a multicomponent structure (motivational interviewing, health coaching, goal setting, and progress monitoring), support behavior change with healthier eating and more frequent exercise [20]. Unfortunately, few studies have tested dietary and physical activity interventions using real-time mHealth to enhance lifestyle self-management of care for KRs [7]. Our proposed study seeks to shift the clinical paradigm for promoting diet intake and physical activity from using education alone and self-reporting to providing a powerful combination of the mHealth app and health coaching skills (setting goals, providing ongoing feedback, and self-monitoring behaviors). Therefore, we developed the Technology, Application, Self-Management for Kidney (TASK) intervention. The TASK intervention is a home-based system-level behavioral intervention for healthy eating and physical activity based on personal routines linked to the environment while monitoring goal attainment, using electronic feedback from mobile health technology.

The purpose of our study was [1] to test the feasibility of (recruitment, retention, and adherence) for using the TASK intervention in post-kidney transplant recipients (≥ 18 years of age) at baseline, 4, 8, and 12 weeks; and [2] estimate the preliminary effects of the TASK intervention in producing change over time for BP, weight, fruits/vegetable intake, fiber intake, sodium intake, self-efficacy to exercise, and perceived stress.

## Methods

### *Study design*

We utilized a 12-week feasibility study using a pre/posttest design. A Midwest University Institutional Review Board approved the study (approval number 2020B0261), and participants gave informed, verbal consent via the telephone to the research assistant (RA).

### Sample

We recruited participants who received care at a Midwest Medical Center Kidney Transplant Clinic or attended a Midwest transplant support group over four months using a convenience sampling strategy. Eligibility criteria for this study consisted of KRs, age 18 years or older, not on dialysis, the ability to speak and hear English, and possession of a smartphone capable of accessing and downloading mobile applications (apps), Wi-Fi, or Internet access. Participants were not eligible for this study if they were participating in a weight loss or structured exercise program or could not pass a brief cognitive test [21].

### Sample size

As expected for a pilot study, the sample size did not have adequate power to detect within-group differences of Cohen’s *d* < 1.0. Therefore, we did not rely solely on statistical significance. Instead, we also interpreted results based on point estimates, precision (e.g., 95% CI) of the estimates, as well as their clinical significance.

### Setting

We conducted the study remotely in the participant’s home using the Zoom platform. No physical contact was made with participants.

### Procedure for the TASK intervention

During the baseline session, participants received instruction from the RA on downloading the Lose-It^©^ app, Health Mate^©^ app, and Fitbit^©^ app virtually using Zoom. The research team (principal investigator and RAs) set up the apps with Gmail accounts with unique, unidentifiable codes developed by the research team. Wi-Fi-connected weight scales and BP cuffs were supplied for weight and BP monitoring. We taught participants how to synchronize the data from the Wi-Fi scale and BP cuff to the Lose-It^©^ app. The Health Mate^©^ app and Fitbit^©^ app sync data from the wireless BP cuff and weight scale automatically to the Lose-It^©^ app, where all the data was stored for food intake, weight, and physical activity. In addition, participants received instructions on how to retrieve data from their Lose-It^©^ app. The participants were instructed by the RA ON how to enter their daily dietary intake and physical activity for 12 weeks. Finally, participants performed a return demonstration to confirm that they could record their daily dietary intake, physical activity, weight, and BP using the Lose-It^©^ app.

Our TASK intervention was adapted from the Plan-Do-Study-Act Model [22]. The intervention began with the development of a “Plan” (individual goals for dietary intake and minutes of physical activity), and the participant identified possible ways (personalized solutions based on their everyday routines) to achieve daily goals. For the “Do” component, participants incorporated their personalized solutions into existing routines. The “Study” component enabled the participants to evaluate their dietary and physical activity goal progress with visual feedback (graphs) from the Lose-It^©^ app. The “Act” phase enabled the participants to evaluate the personalized-system solution and determine the achievement of the dietary and physical activity goals.

During the session weeks 1–12, the participant completed four steps of the Plan-Do-Study-Act Model with the RA via Zoom. In Step 1, the participant placed the goals (dietary and physical activity) into their Lose-It^©^ app, via smartphone, for goal attainment. During Step 2, the participant and the RA reviewed possible ways to incorporate their personalized solution into existing routines. For example, they packed a healthy lunch and gym bag with exercise clothing the night before. Each week in Step 3, the RA reviewed with the participant the electronic report generated from the Lose-It^©^ for the number of days they achieved their daily goals. Lastly, in Step 4, the RA and the participant evaluated the personalized-system solution and determined the achievement of the dietary and physical activity goals.

### Data collection

#### Variables collected

The primary aim was to evaluate the feasibility of recruitment (meeting recruitment targets), retention (the number of participants to drop the study), and adherence to the intervention (percent to adhere to logging daily dietary intake and physical activity). The second aim was to estimate the preliminary effects of the TASK intervention in producing change over time for BP, weight, fruits/vegetables intake, fiber intake, sodium intake, self-efficacy to exercise, and perceived stress. The participants measured their BP daily using a wireless Wi-Fi BP cuff, which they applied to the upper arm. Participants recorded their BP each day at the same time, seated in a chair with their legs uncrossed and feet flat on the floor, using a wireless Wi-Fi BP cuff. Each day, data from the wireless cuff was synced directly from the participant’s Lose-It^©^ app to the premium password-protected Lose-It^©^ database called Ascend. The participant measured their weight each morning, at the same time of day, with no clothes, using a wireless Wi-Fi weight scale. Each day, data from the wireless weight scale was synced directly to the Ascend database.

We measured fruit and vegetable intake using the Block Fruit-Vegetable-Fiber Screener. This brief screening tool includes seven questions about fruit and vegetable intake and three questions about foods high in fiber, magnesium, and potassium. The Block Fruit-Vegetable-Fiber Screener has demonstrated high reliability (Spearman* r*-value of 0.71) [23]. The percent of fat intake and carbohydrate intake was calculated and recorded each day by the Lose-It^©^ app based on the food that was recorded within the app. Self-efficacy to exercise was measured by the survey called the Self-Efficacy for Exercise (SEE) Scale. The SEE scale is a nine-item questionnaire using a Likert Scale to rate feelings of stress from 0 “not confident” to 10 “very confident.” The SEE has demonstrated excellent internal consistency (*α* = 0.92) for adults living with chronic disease [24] Lastly, we measured the perceived stress using the Perceived Stress Scale (PSS) [25]. The PSS is a 10-item questionnaire using a Likert Scale to rate feelings of stress from 0 “never” to 4 “very often.” The PSS has demonstrated excellent test–retest reliability (intraclass correlation coefficient = 0.954) and internal consistency (Cronbach’s alpha = 0.810) with adults who have chronic disease [26].

#### Data collection

The RA entered all study data directly into REDCap. Similarly, mobile data collected by the Lose-It^©^ app was secured in the Lose-It^©^ password-protected database, Ascend.

### Statistical analysis

Descriptive statistics summarized the sample characteristics at baseline. Descriptive statistics were also used to analyze the dietary recording adherence, physical activity recording, and all other outcome variables (e.g., systolic BP) at baseline, Week 4, Week 8, and Week 12. Mixed-effects logistic regression models were used to examine the associations between participants’ dietary intake logging adherence, physical activity logging adherence, study weeks (time), participants’ weight, and BP. We applied paired *t*-tests and McNemar’s test to compare the outcomes at Week 4, Week 8, and Week 12 with their values at baseline; 95% confidence intervals and effect sizes as well as *p*-values were reported for continuous outcomes; and *p*-values were reported for categorical outcomes. A *p*-value less than 0.05 was deemed to be significant, and all the analyses were conducted in R 4.1.2.

### Treatment fidelity

We used a training manual developed and implemented successfully in our previous study [11]. The research assistants (RAs) attended four training sessions to review the Lose-It^©^ app, Health Mate^©^ (wireless BP Cuff), and Fitbit^©^ (wireless scale) app download setup using recorded weight from the wireless scale and BP from the wireless cuff monitoring. The RAs reviewed case studies virtually for how to communicate with participants. The RAs role-played with simulated participants for the setup of equipment and how to virtually collect the data using REDCap. Following each training session, the RAs completed a debriefing session to identify areas requiring improvement.

The RAs ensured the protocol delivery by using a checklist of all the steps needed to complete each session and a log of the time taken to confirm equivalent treatment dose across all sessions for both groups. Participants were required to demonstrate their ability to use the Lose-It^©^ app, wireless BP cuff, and scale. The RAs reviewed with the participant their progress via the Lose-It^©^ app progress report (electronic report) and their success in using the intervention.

## Results

### Sample

The mean age of the participants was 59.4 ± 10.7 years. The majority of the participants were white and not Hispanic. The greatest portion of participants reported receiving an associate degree or higher, earning an income $65,000 or less per year, having at least two people living in the household, and not being employed. Most participants received their kidney from a deceased donor (70%) and took the immunosuppressant mycophenolate (60%, *n* = 12). The participants lived an average of 50 miles from the transplant center, and 30% (*n* = 6) of the participants lived in a rural area (Table 1).

**Table 1:** Demographics for kidney transplant recipients (*N* = 20) using TASK intervention

Variables	Mean ± SD or *N* (%)
**Age**	59.45 ± 10.70
**Gender**	
Male	10 (50%)
Female	10 (50%)
**Ethnicity**	
Hispanic/Latino	1 (5%)
Not Hispanic/Latino	19 (95%)
**Race**	
African American or Black	6 (30%)
White	14 (70%)
**Education**	
High School Diploma or GED	4 (20%)
Associate’s or Technical	5 (25%)
Bachelor’s	7 (35%)
Master’s	4 (20%)
**Income**	
Under $20,000	1 (5%)
$20,000–$45,000	5 (25%)
$46,000–$65,000	5 (25%)
$91,000–$125,000	6 (30%)
$126,000 +	3 (15%)
**Number of household members**	
1	3 (15%)
2	11 (55%)
3	4 (20%)
4	2 (10%)
**Caregiver**	
Self	18 (90%)
Spouse or partner	1 (5%)
Parent or legal guardian	1 (5%)
**Type of transplant**	
Deceased donor	14 (70%)
Living donor	6 (30%)
**Number of kidney transplants**	
1	18 (90%)
2 +	2 (10%)
**Employment**	
Full-time employment	5 (25%)
Not employed	9 (45%)
Retired	6 (30%)

### Recruitment and retention

**Fig. 1:**
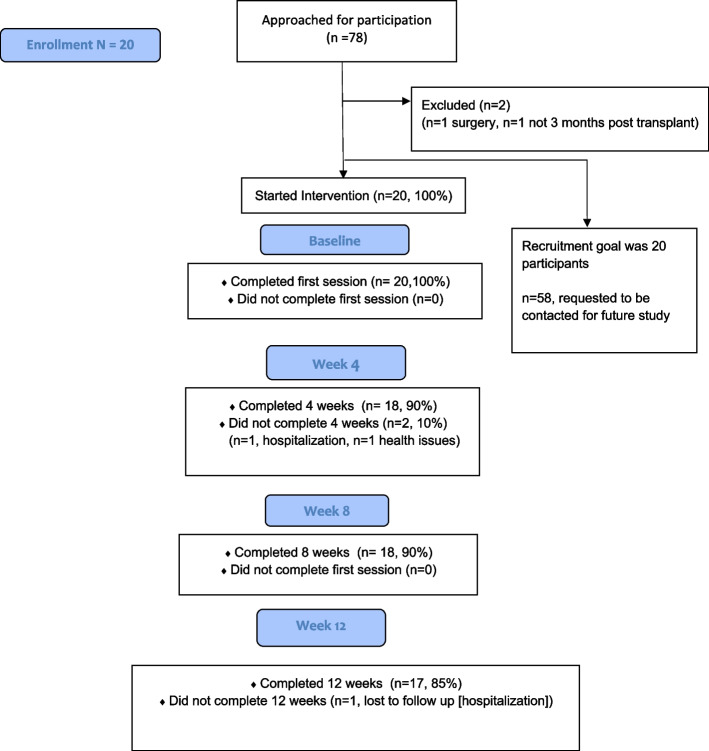
The attrition rate of participants not completing the study

We met our target goal of recruiting 20 participants. However, two participants dropped out of the study due to hospitalization and health issues. One additional participant was lost to follow-up after Week 12. We found a 15% attrition rate of participants not completing the study (Fig. 1).

### Adherence

The adherence rate over the 12 weeks among the study completers for recording daily food intake via the Lose-It^©^ app ranged from 83 to 94%. The adherence rate among the study completers for recording daily physical activity via the Lose-It^©^ app ranged from 17 to 33% over the 12 weeks. We found a significant association between adherence (logging dietary and physical activity) and study weeks or time (OR, 0.79, [95% CI, 0.63, 0.99], *p* = 0.04). As time passed, the participants were less adherent to logging both food intake and physical activity (Tables 2 and 3).

**Table 2:** Summary of adherence for recording daily food intake and physical activity

Week	Food intake	Physical activity
Week 1	17 (94%)	5 (28%)
Week 2	17 (94%)	5 (28%)
Week 3	17 (94%)	6 (33%)
Week 4	16 (89%)	5 (28%)
Week 5	16 (89%)	3 (17%)
Week 6	16 (89%)	3 (17%)
Week 7	15 (83%)	5 (28%)
Week 8	16 (89%)	4 (22%)
Week 9	17 (94%)	6 (33%)
Week 10	16 (89%)	5 (28%)
Week 11	15 (83%)	5 (28%)
Week 12	15 (83%)	3 (17%)

**Table 3:** Associations between recording (food intake and physical activity) adherence and time, weight, SBP, and DBP

Variables	Food intake adherence		Physical adherence	
	**Odds ratio (95% CI)**	***p*** **-value**	**Odds ratio (95% CI)**	***p*** **-value**
**Week**	0.79 (0.63, 0.99)	0.042	0.94 (0.81, 1.10)	0.457
**Weight (scaled)**	0.04 (0.00, 1.52)	0.083	0.58 (0.02, 17.77)	0.753
**SBP (scaled)**	23.39 (0.10, 5711.87)	0.261	1.29 (0.01, 239.11)	0.924
**DBP (scaled)**	0.24 (0.00, 15.23)	0.501	1.40 (0.02, 122.95)	0.882

### Blood pressure and weight

Improvement in the average for both systolic and diastolic BP was observed over the 12 weeks. No significant improvements were observed in BP from baseline to 12 weeks (Table 2). Reduction in weight was observed from baseline (190.1 ± 44.6) to 12 weeks (186.6 ± 47). We found a significant difference and a medium effect size in weight loss among the participants from baseline compared to 12 weeks ([95% CI, 0.88–9.08], *d* = 0.62, *p* = 0.02) (Table 4).

**Table 4:** Estimates of the effects of the TASK intervention between Week 4, Week 8, Week 12, and baseline

Variables	Mean ± SD or *N* (%)				Week 4 vs. baseline			Week 8 vs. baseline			Week 12 vs. baseline		
	**Baseline**	**Week 4**	**Week 8**	**Week 12**	**Difference (95% CI)**	**Effect size**	***p*** **-value**	**Difference (95% CI)**	**Effect size**	***p*** **-value**	**Difference (95% CI)**	**Effect size**	***p*** **-value**
**Systolic blood pressure**	134.28 ± 27.46	137.33 ± 20.92	129.89 ± 26.22	128.47 ± 18.99	3.06 (− 4.90, 11.01)	0.19	0.429	− 4.39 (− 12.24, 3.46)	− 0.28	0.255	− 7.00 (− 15.55, 1.55)	− 0.42	0.102
**Diastolic blood pressure**	80.39 ± 16.67	78.44 ± 10.02	76.28 ± 11.27	76.94 ± 11.83	− 1.94 (− 8.99, 5.10)	− 0.14	0.568	− 4.11 (− 10.59, 2.37)	− 0.32	0.198	− 3.76 (− 10.08, 2.55)	− 0.31	0.225
**Weight**	190.11 ± 44.57	189.48 ± 44.84	182.88 ± 38.36	186.61 ± 47.02	− 0.62 (− 2.23, 0.98)	− 0.19	0.425	− 7.22 (− 18.78, 4.34)	− 0.31	0.205	− 4.98 (− 9.08, − 0.88)	− 0.62	0.020
**Fruits/vegetables intake**							0.371			0.724			0.289
2 servings or less	11 (58%)	8 (44%)	9 (50%)	7 (41%)	-	-		-	-		-	-	
3 servings or more	8 (42%)	10 (56%)	9 (50%)	10 (59%)	-	-		-	-		-	-	
**Fiber intake**							1.000			1.000			1.000
20 mg or less	16 (84%)	15 (83%)	15 (83%)	14 (78%)	-	-		-	-		-	-	
21 mg or more	3 (16%)	3 (17%)	3 (17%)	4 (22%)	-	-		-	-		-	-	
**Sodium intake**	2147.52 ± 806.85	1714.18 ± 645.98	1662.09 ± 472.95	1878.60 ± 1306.05	− 433.34 (− 765.40, − 101.27)	− 0.67	0.014	− 485.43 (− 876.00, − 94.86)	− 0.64	0.018	− 326.83 (− 1202.46, 548.80)	− 0.20	0.439
**Percent of fat intake**	29.59 ± 14.17	28.49 ± 9.07	28.51 ± 7.59	25.98 ± 6.44	− 1.10 (− 6.96, 4.76)	− 0.10	0.696	− 1.08 (− 7.72, 5.56)	− 0.08	0.734	− 4.15 (− 11.53, 3.23)	− 0.30	0.249
**Percent of carbohydrate intake**	32.69 ± 9.42	32.65 ± 8.76	36.12 ± 11.11	33.24 ± 10.17	− 0.04 (− 5.25, 5.18)	0.00	0.989	3.43 (− 2.83, 9.69)	0.28	0.263	0.44 (− 5.80, 6.68)	0.04	0.882
**Self-efficacy to exercise (SEE)**	60.75 ± 23.35	71.00 ± 18.69	71.17 ± 17.67	80.71 ± 17.68	9.61 (− 0.55, 19.77)	0.47	0.062	9.78 (− 2.01, 21.56)	0.41	0.098	17.06 (6.51, 27.60)	0.83	0.003
**Perceived Self-Stress Scale (PSS)**	21.65 ± 3.88	17.56 ± 5.03	18.00 ± 4.79	18.82 ± 5.27	− 3.56 (− 5.65, − 1.46)	− 0.84	0.002	− 3.11 (− 5.07, − 1.15)	− 0.79	0.004	− 2.53 (− 4.90, − 0.16)	− 0.55	0.038

### Dietary intake

We asked the participants if they had ever learned about “My Plate” as part of their education after the transplant, and 90% (*n* = 18) of participants reported that they had never seen it or received any information about eating healthfully. At baseline, over half (58%) of the sample consumed two or fewer servings of fruits and vegetables daily. After 12 weeks, more than half (59%) of the sample consumed three or more servings per day of fruits and vegetables. Fiber intake remained low among most participants over the 12 weeks. The average sodium intake was high among this sample, but sodium intake decreased at Week 4 by 433.3 mg ([95% CI, 101.3–765.4], *p* = 0.014) and Week 8 by 485.4 mg ([95% CI, 94.86–876.0], *p* = 0.018). We found no significant reduction, however, in sodium intake from baseline to Week 12. Additionally, we found no significant difference from baseline to 12 weeks in the percent of fat and carbohydrate intake (Table 4).

### Self-efficacy to exercise (ESF)

After 12 weeks of using the intervention, we found a significant increase in self-efficacy to exercise (baseline mean, 61; 12 weeks mean, 81 (MD = 20 [95% CI, 6.51, 27.6], *d* = 0.83, *p* = 0.003) (Table 4).

### Perceived Self Stress Scale (PSS)

Participants reported less perceived stress over time at 4, 8, and 12 weeks. After 12 weeks of using the intervention, we found a significant decrease in perceived stress (baseline mean, 22; 12 weeks mean, 19 (MD = 3 [95% CI, 0.16, 4.90], *d* = 0.55, *p* = 0.04) (Table 4).

## Discussion

The primary aim of this study was to determine the feasibility of recording food intake and physical activity using mHealth technology and teaching kidney recipients’ health coaching skills. We found that kidney recipients were receptive to using health coaching skills to achieve individualized goals for dietary intake and minutes of physical activity. These health coaching skills included helping the participants identify personalized solutions based on their normal routines to achieve their daily goals and studying their progress to evaluate their dietary and physical activity goal progress with visual feedback (graphs) from the Lose-It^©^ app. We met our recruitment goal and had additional potential kidney recipients’ requests to participate in future studies. Retention was high throughout the study. Only three participants did not complete the study because of hospitalizations and illness. Adherence rates for recording daily dietary intake were higher than for physical activity throughout the study. However, over time, participants were less adherent to logging both food intake and physical activity. This finding concurs with another study that used mobile dietary monitoring to track diet intake. This mobile dietary monitoring study found that participants’ dietary tracking declined over time and that fewer than half of the sample still recorded their dietary intake after 10 weeks [27]. A suggestion for future studies is to include boosters or reminders for logging diet and physical activity. Also, our participants did not have a device to provide ongoing monitoring of physical activity. Another suggestion would be to provide an activity tracker.

For our secondary aims, we wanted to estimate the effects of intervention in producing change over time for BP, weight, fruits/ intake, fiber intake, sodium intake, self-efficacy to exercise, and perceived stress. However, due to the small sample size, it is important to use caution when interpreting the preliminary estimate of the effects for the study. Although we did find overall improvements in BP, fruits/vegetables intake, fiber intake, and sodium intake, we did not find any significant change effect over the 12 weeks. A previous study conducted with chronic kidney disease patients found telehealth coaching was safe and resulted in weight loss over time, but did not improve BP [28]. One mediating factor to consider for the sodium intake being higher at 12 weeks compared to Week 4 and Week 8 is that many participants completed the study between Thanksgiving and Christmas holidays. Holiday events may have resulted in participants consuming food higher in sodium. This finding is similar to other studies that used lifestyle-coaching approaches to improve adherence for physical activity [29] and dietary tracking [30] where participants reported more barriers to health behaviors during the holidays [29, 30]. We did find that many participants requested more recipes to incorporate low-sodium food and fruits and vegetables into their diet. In the future, we plan to incorporate these types of recipes into future studies.

Interestingly, we found a large effect in self-efficacy to exercise from baseline to 12 weeks. A similar study found that self-efficacy to exercise significantly increased in cardiopulmonary patients after 12 weeks of wearing a mobile device to record physical activity [31]. However, a recent meta-analysis suggested that future studies are needed to explore the underlying mechanism of engagement for using the mobile technology, thereby resulting in self-efficacy to exercise [32].

In addition, we found a medium effect for weight reduction and perceived stress change over the 12 weeks. Mounting evidence indicates that individuals across the lifespan who participate in regular physical activity report lower levels of perceived stress [33–35]. Physical activity as a stress management technique is a preventative mechanism for perceived stress [36].

Weight reduction was observed in another study using mHealth technology to enhance self-monitoring. This study was conducted by Burke et al. [37] and found that mHealth technology played a crucial role in self-monitoring behaviors, resulting in weight loss. However, a randomized controlled trial conducted by Henggeler et al. [38] in 2018 tested intensive nutrition intervention (individualized nutrition and exercise counseling, 12 dietitian visits, and three exercise physiologist visits over 12 months) in KRs compared to standard nutrition care (four dietitian visits) 1 month after transplant. The study results indicated that both groups gained weight, and kidney recipients did not benefit from the intensive nutrition intervention in the first year after transplant [38]. The Henggeler et al. [38] study, however, did not use mobile technology to track dietary intake and physical activity. Further studies are needed to investigate nutrition interventions using mobile technology to control weight gain in kidney transplant recipients greater than 1-year posttransplant.

Blood pressure reduction was demonstrated at weeks 4 and 8 compared to baseline. Earlier studies involving self-monitoring of BP resulted in lowering BP [39–41]. McGillicuddy et al. [42] tested BP control using mHealth in kidney transplant patients and observed a relative reduction in systolic BP in the group that used mHealth for monitoring compared to the group that did not. It is widely known that weight gain may increase BP. Therefore, our study may have clinically meaningful results, as previous studies have found that a 3 kg (6.6 pounds) weight loss reduces BP [4, 43].

This study has limitations, although the data were promising. First, we recruited from a single-site transplant center and obtained a small sample in which most participants were white. Secondly, the time of participation, which was during the holiday seasons between Thanksgiving, Christmas, and New Year, might have affected the participants’ dietary intake. Thirdly, a longitudinal study with a longer period might have been more beneficial in assessing weight and BP change over time. Lastly, we did not collect information about the length of time since transplant. It is possible that many of our participants were greater than 1 year posttransplant and were more stabilized. Future studies should explore the efficacy and effectiveness of the intervention using a longitudinal, randomized, controlled trial design for posttransplant kidney recipients greater than 1 year. In addition, researchers should consider collecting kidney function data, such as renal function. Future studies should also explore behavioral factors that contribute as facilitators or barriers to using the intervention, for example, best methods for recording dietary intake and best techniques on how to capture missing dietary intake data.

## Conclusions

This study has taken the first step in utilizing an intervention called TASK (mHealth apps + health coaching skills) in kidney transplant recipients. One should interpret our study’s results with caution due to the small sample size and lack of adequate statistical power. Nevertheless, our findings will aid in developing a large, fully powered RCT to address the health benefits of dietary and physical activity tracking using mobile technology and health coaching. In summary, we found that the TASK intervention to enhance self-management for physical activity and dietary intake was feasible and resulted in weight control, increased self-efficacy to exercise, and decreased stress. These findings are significant given the number of recipients who gain weight after a kidney transplant. A multicomponent intervention such as the TASK intervention may facilitate care self-management after a kidney transplant.

## Data Availability

Yes, upon written request.
