# Correction: The feasibility of Technology, Application, Self-Management for Kidney (TASK) intervention in post-kidney transplant recipients using a pre/posttest design

**DOI:** 10.1186/s40814-024-01444-0

**Published:** 2024-01-10

**Authors:** Tara O’Brien, Karen Rose, Brian Focht, Noor Al Kahlout, Tad Jensen, Kenzie Heareth, Uday Nori, Reem Daloul

**Affiliations:** 1 The Ohio State University College of Nursing, Newton Hall, 1585 Neil Ave, Columbus, OH 43210 USA; 2 The Ohio State University College of Education and Human Ecology, 152 PAES, 305 Annie and John Glenn Ave, Columbus, OH 43210 USA; 3 The Ohio State University College of Medicine, 300 West 10th Avenue Suite 1150, Columbus, OH 43210 USA; 4 Division of Nephrology, Kidney and Pancreas Transplant Program, Allegheny General Hospital, Erie, PA 16505 USA

**Correction** **:** **Pilot Feasibility Stud 9, 190 (2023)**

**https://doi.org/10.1186/s40814-023-01417-9**

Following publication of the original article [1], the authors identified an error in the author name of Uday Nori.

The incorrect author name is: Uday Noir

The correct author name is: Uday Nori

The author group has been updated above and the original article [1] has been corrected.
